# Tembusu Virus in Ducks, China

**DOI:** 10.3201/eid1710.101890

**Published:** 2011-10

**Authors:** Zhenzhen Cao, Cun Zhang, Yuehuan Liu, Weicheng Ye, Jingwen Han, Guoming Ma, Dongdong Zhang, Feng Xu, Xuhui Gao, Yi Tang, Shaohua Shi, Chunhe Wan, Chen Zhang, Bin He, Mengjie Yang, Xinhao Lu, Yu Huang, Youxiang Diao, Xuejun Ma, Dabing Zhang

**Affiliations:** Key Laboratory of Zoonosis of Ministry of Agriculture, Beijing, People’s Republic of China (Z. Cao, G. Ma, Dongdong Zhang, Dabing Zhang);; China Agricultural University, Beijing (Z. Cao, G. Ma, Dongdong Zhang, Dabing Zhang);; Zhejiang Academy of Agricultural Sciences, Hangzhou, People’s Republic of China (Cun Zhang, W. Ye);; Beijing Academy of Agriculture and Forestry Sciences, Beijing (Y. Liu, J. Han);; Beijing University of Agriculture, Beijing (F. Xu);; Shandong Agricultural University, Taian, People’s Republic of China (X. Gao, Y. Tang, Y. Diao);; Fujian Academy of Agricultural Sciences, Fuzhou, People’s Republic of China (S. Shi, C. Wan, Y. Huang);; Chinese Center for Disease Control and Prevention, Beijing (Chen Zhang, B. He, M. Yang, X. Ma);; Yuyao Municipal Institute of Poultry Disease, Yuyao, People’s Republic of China (X. Lu)

**Keywords:** Flavivirus, duck flavivirus, duck hemorrhagic ovaritis, Tembusu virus, viruses, China, dispatch

## Abstract

In China in 2010, a disease outbreak in egg-laying ducks was associated with a flavivirus. The virus was isolated and partially sequenced. The isolate exhibited 87%–91% identity with strains of Tembusu virus, a mosquito-borne flavivirus of the Ntaya virus group. These findings demonstrate emergence of Tembusu virus in ducks.

From June through November 2010 in the People’s Republic of China, a disease characterized by a sudden onset was observed on many egg-laying and breeder duck farms. Egg production in affected ducks dropped severely within 1–2 weeks after disease onset. Other consistent signs included acute anorexia, antisocial behavior, rhinorrhea, diarrhea, ataxia, and paralysis. Rate of illness was usually high (up to 90%), and mortality rates varied from 5% to 30%. From affected ducks we isolated and identified a Tembusu virus (TMUV).

## The Study

During the outbreak, we examined 11 diseased ducks (7 Pekin ducks, 3 Cherry Valley Pekin ducks, and 1 Shaoxing duck) from 5 duck farms in 4 provinces. At necropsy, viscera samples (e.g., brain, heart, liver, spleen, lung, theca folliculi) were collected and placed in 10% buffered formalin. Sections were embedded in paraffin and stained with hematoxylin and eosin. The theca folliculi from each duck were also used for virus isolation or detection by PCR.

**Figure 1:**
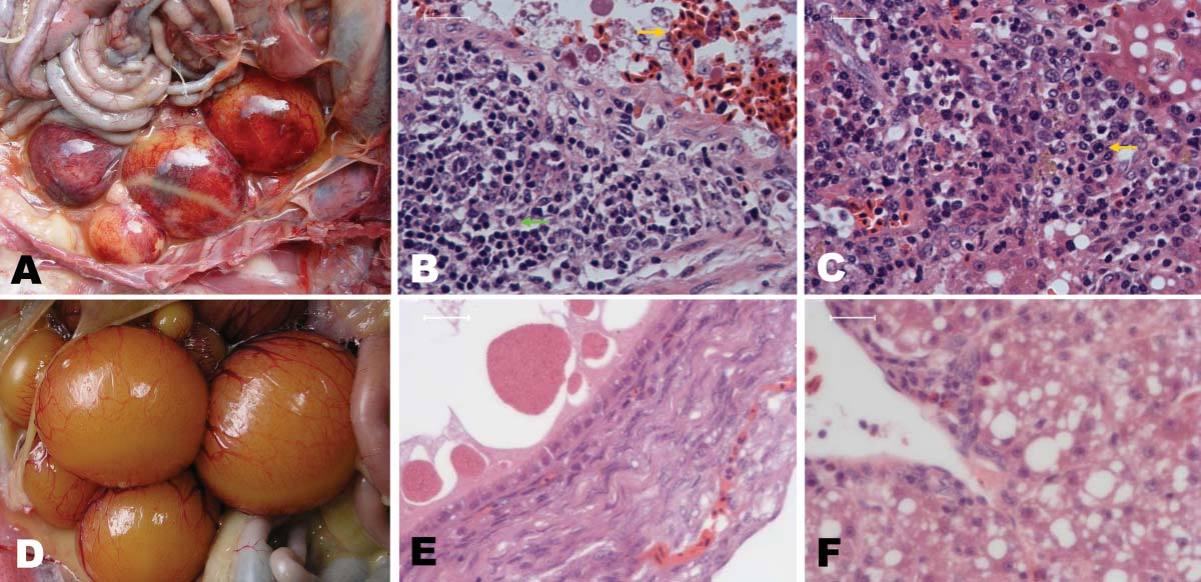
Pathologic changes in diseased Pekin ducks. A) Ovary with hyperemia, hemorrhage, and distortion. B) Ovary with hemorrhage (gold arrow), macrophage and lymphocyte infiltration and hyperplasia (green arrow). C) Liver with interstitial inflammation in the portal area (gold arrow). D and E) Ovaries from healthy ducks. F) Liver from healthy duck. A, C) Original magnification ×40; B, C, E, F) scale bars = 90 μm.

The main pathologic changes observed consistently in almost all diseased ducks were found in the ovaries: hyperemia, hemorrhage, degeneration, distortion, macrophage and lymphocyte infiltration, and hyperplasia; in the liver, interstitial inflammation was found in the portal area (Figure 1, panels A–C). On the basis of these changes, the disease was designated duck hemorrhagic ovaritis.

After samples underwent several rounds of screening and identification attempts, we concluded that a new virus infection was the most likely cause of the disease. One virus strain, designated YY5, was isolated from the Shaoxing duck after inoculation of clarified theca folliculus suspension into the allantoic cavities of 9-day-old specific pathogen–free chicken embryos. The embryos died 72–120 hours after inoculation, and severe cutaneous hemorrhages were observed.

Because eastern equine encephalitis (EEE) virus has been shown to cause paralysis in Pekin ducks (*1*), we performed EEE virus–specific nested reverse transcription PCR (RT-PCR) (*2*) to detect the isolate. RNA was extracted by using the TranZol RNA Extraction Kit (TransGen Biotech, Beijing, China). The primer pairs used to amplify the E2 gene of EEE virus (EEE-4 and cEEE-7, EEE-5 and cEEE-6) have been described (*2*). A clear PCR product (268 bp) resulted and was then sequenced; the deduced amino acid sequence was compared with other sequences by using a BLASTP (http://blast.ncbi.nlm.nih.gov/Blast.cgi) search in GenBank. Unexpectedly, a 221-nt sequence (GenBank accession no. HQ641388) was shown to encode the nonstructural (NS) 1 protein of flavivirus, which exhibited 73%–85% identity to flaviviruses in the Ntaya virus and Japanese encephalitis virus groups, such as Bagaza virus (GenBank accession no. ACG60714 [*3*],) and St. Louis encephalitis virus (GenBank accession no. ABN11829 [*4*]). The result demonstrated the possible presence of a flavivirus in ducks.

To further confirm flavivirus as the causative agent of duck hemorrhagic ovaritis, we used PCR to test the isolate and clinical samples with forward primer Usu5454f (5′-ATGGATGAAGCYCATTTCAC-3′) (*5*) and a newly designed reverse primer 5861R (5′-CCAAAGTTGGCYCCCATCTC-3′). The primers were located in the conserved regions of the NS3 sequences of Bagaza virus, St. Louis encephalitis virus, and Usutu virus (*3*–*5*) and were predicted to produce an ≈400-bp amplicon. The reaction conditions were as follows: 5 min at 94°C; followed by 38 cycles of denaturation at 94°C for 40 s, annealing at 47°C for 35 s, and extension at 72°C for 1 min; and a final extension of 72°C for 10 min. RT-PCR was optimized by using the following controls, including nucleic acids extracted from theca folliculi of healthy Pekin ducks: avian influenza virus, Newcastle disease virus, egg drop syndrome virus, anatid herpesvirus 1, Muscovy duck parvovirus, goose parvovirus, duck reovirus, goose reovirus, duck hepatitis A virus, duck astrovirus, duck circovirus, and goose hemorrhagic polyomavirus.

All 11 theca folliculus samples and the isolate were positive for flavivirus by RT-PCR, which was confirmed by amplicon sequencing. The 367-nt sequence (GenBank accession no. HQ641389) of part of the NS3 genomic region obtained from the isolate was 66%–77% identical to the corresponding sequence of viruses in the Ntaya virus and Japanese encephalitis virus groups. The amplicon sequences from the 11 theca folliculus samples shared 98%–100% identity with the YY5 isolate. PCR testing of another 52 samples from diseased ducks from the 4 provinces detected flavivirus-specific RNA in 29 samples. Overall, 40 (63.5%) of 63 samples were positive for flavivirus. Flavivirus-specific RNA was most frequently detected in theca folliculi, followed by intestinal mucosa, uterus, spleen, trachea, cloaca (swab), and liver (Table).

**Table:** Flavivirus detection in 63 samples from diseased ducks, 4 provinces, People’s Republic of China, 2010

Sample	No. (%) flavivirus positive	No. flavivirus negative
Theca folliculi, n = 15	14 (93.3)	1
Intestinal mucosa, n = 4	3 (75.0)	1
Uterus, n = 7	5 (71.4)	2
Spleen, n = 9	6 (66.7)	3
Trachea, n = 2	1 (50.0)	1
Cloacal swab, n = 17	8 (47.1)	9
Liver, n = 9	3 (33.3)	6

Subsequently, the YY5 isolate was injected intramuscularly into nine 55-week-old Pekin ducks and ten 30-week-old Shaoxing ducks. At day 4 postinoculation, the pathologic changes were reproduced in these experimentally infected ducks. The flavivirus RNA was detected by NS3-based RT-PCR, and the virus was again isolated from theca folliculi.

**Figure 2:**
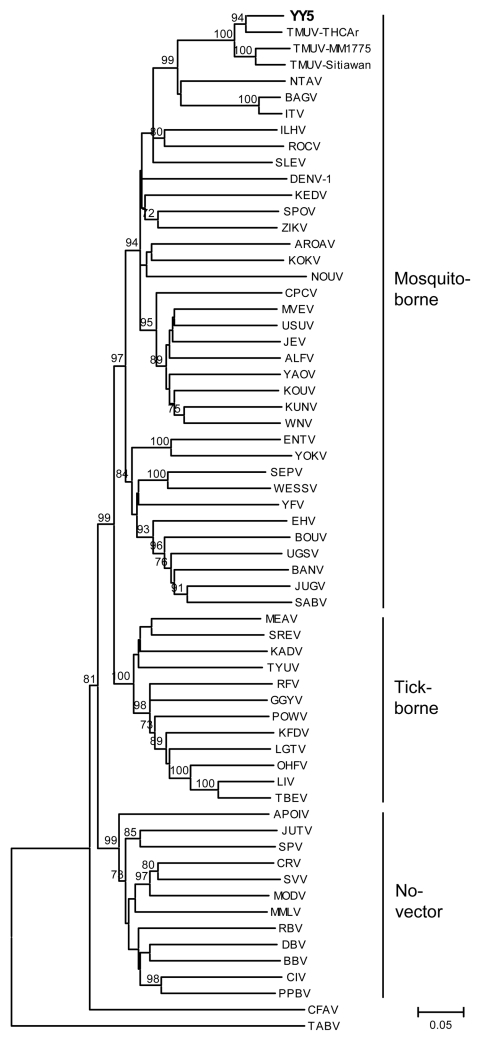
Phylogenetic analysis of isolate YY5 (in **boldface**) from an ill Shaoxing duck in the People’s Republic of China and selected other flaviviruses obtained by using an ≈1-kb nt sequence in the nonstructural 5 genomic region. The tree was constructed by the neighbor-joining method of MEGA (*7*). Numbers at nodes indicate bootstrap percentages obtained after 1,000 replicates; only bootstrap values >70% are shown. Scale bar indicates genetic distance. The sequences used in the phylogenetic analysis are as follows: AF013374, Gadgets Gully virus (GGYV); AF013385, Kyasanur Forest disease virus (KFDV); M86650, Langat virus (LGTV); Y07863, louping ill virus (LIV); AF013393, Omsk hemorrhagic fever virus (OHFV); L06436, Powassan virus (POWV); AF013398, Royal Farm virus (RFV); U27495, tick-borne encephalitis virus (TBEV); AF013380, Kadam virus (KADV); AF013386, Meaban virus (MEAV); AF013403, Saumarez Reef virus (SREV); AF013410, Tyuleniy virus (TYUV); AF013362, Aroa virus (AROAV); NC_001477, dengue virus 1 (DENV-1); AF013382, Kedougou virus (KEDV); AF013367, Cacipacore virus (CPCV); U15763, Japanese encephalitis virus (JEV); AF013384, Koutango virus (KOUV); AF013360, Alfuy virus (ALFV); AF013389, Murray Valley encephalitis virus (MVEV); AF013416, St. Louis encephalitis virus (SLEV); AF013412, Usutu virus (USUV); M12294, West Nile virus (WNV); D00246, Kunjin virus (KUNV); AF013413, Yaounde virus (YAOV); AF013383, Kokobera virus (KOKV); AF013363, Bagaza virus (BAGV); AF013376, Ilheus virus (ILHV); AF013397, Rocio virus (ROCV); AF013377, Israel turkey meningoencephalomyelitis virus (ITV); AF013392, Ntaya virus (NTAV); AF013408, Tembusu virus strain MM1775 (TMUV-MM1775); AF013409, TMUV strain THCAr (TMUV-THCAr); AB026994, TMUV strain Sitiawan (TMUV-Sitiawan); AF013415, Zika virus (ZIKV); AF013406, Spondweni virus (SPOV); L40951, Banzi virus (BANV); AF013364, Bouboui virus (BOUV); AF013372, Edge Hill virus (EHV); AF013378, Jugra virus (JUGV); AF013400, Saboya virus (SABV); AF013404, Sepik virus (SEPV); AF013411, Uganda S virus (UGSV); EU707555, Wesselsbron virus (WESSV); X03700, yellow fever virus (YFV); AF013373, Entebbe bat virus (ENTV); AF013414, Yokose virus (YOKV); AF013361, Apoi virus (APOIV); AF013370, Cowbone Ridge virus (CRV); AF013379, Jutiapa virus (JUTV); AF013387, Modoc virus (MODV); AF013401, Sal Vieja virus (SVV); AF013402, San Perlita virus (SPV); AF013365, Bukalasa bat virus (BBV); AF013368, Carey Island virus (CIV); AF013371, Dakar bat virus (DBV); AF013388, Montana myotis leukoencephalitis virus (MMLV); AF013394, Phnom Penh bat virus (PPBV); AF013396, Rio Bravo virus (RBV); M91671, cell fusing agent virus (CFAV); NC_003996, Tamana bat virus (TABV); and EU159426, Nounané virus (NOUV). The nucleotide sequence of isolate YY5 used in the phylogenetic analysis has been deposited in GenBank under accession no. HQ641390.

To investigate the genetic relationship of the isolate with flaviviruses, we obtained the genomic sequence of a 1,035-bp segment at the 3′ terminus of the NS5 gene (GenBank accession no. HQ641390) from strain YY5 by RT-PCR and primers FU1 and cFD3 as described (*6*). Phylogenetic analysis showed that YY5 was more closely related to TMUV than to other flaviviruses (Figure 2). Comparative sequence analysis showed that YY5 was 87%–91% identical to different strains of TMUV; therefore, we classified the flavivirus isolated from ducks as a new genotype of TMUV, a mosquito-borne flavivirus of the Ntaya virus group.

## Conclusions

We have demonstrated the presence of a mosquito-borne flavivirus in ducks. On the basis of criteria for species of the members of the genus *Flavivirus* (*6*) and phylogenetic analysis, we consider the isolate to belong to a new genotype of TMUV.

In this study, we found TMUV-specific RNA in 63.5% samples from diseased ducks in different provinces. In particular, it was found in 14 (93.3%) of 15 theca folliculus samples, suggesting that reproductive tissues may be a major site for viral persistence, replication, or both. Experimental infections further confirmed that TMUV can be reisolated from theca folliculi. These results suggested that TMUV may be the causative agent of duck hemorrhagic ovaritis.

Because TMUV belongs to the mosquito-borne virus cluster of flaviviruses, mosquitoes might be involved in the spread of this virus. Detection of the virus in cloacal swab samples suggests probable horizontal transmission through ingestion or inhalation of feces-contaminated material.

TMUV was originally isolated from mosquitoes of the genus *Culex*, but the disease associated with TMUV infection was not known. However, a chick-origin TMUV isolate, originally named Sitiawan virus, can cause encephalitis and retarded growth in broiler chicks (*8*). In conclusion, this study shows that duck-origin TMUV is highly pathogenic for Pekin ducks, Cherry Valley Pekin ducks, and Shaoxing ducks.
